# Explaining the disability paradox: a cross-sectional analysis of the Swiss general population

**DOI:** 10.1186/1471-2458-12-655

**Published:** 2012-08-15

**Authors:** Bernd Fellinghauer, Jan D Reinhardt, Gerold Stucki, Jerome Bickenbach

**Affiliations:** 1 Swiss Paraplegic Research (SPF), Guido A. Zäch Str. 4, 6207 Nottwil, Switzerland; 2 Seminar für Statistik, ETH Zurich, Rämistr. 101, 8092 Zurich, Switzerland; 3 Department of Health Sciences and Health Policy, University of Lucerne and SPF, Frohburgstr. 3, 4466 Lucerne, Switzerland

**Keywords:** Disability, Human functioning, Perceived health, Impairment, ICF, Graphical models, Activity limitation, Participation restriction

## Abstract

**Background:**

Disability can be broken down into difficulties in different components of functioning such as impairments and limitations in activities and participation (A&P). Previous studies have produced the seemingly surprising result that persons with severe impairments tend to report high quality of life (QoL) including perceived health regardless of their condition; the so-called “disability paradox”. We aim to study the role of contextual factors (i.e. the personal and environmental situation) in explaining the disability paradox.

**Methods:**

The Swiss Health Survey provides information on the perceived health of 18,760 participants from the general population. We construct a conditional independence graph applying random forests and stability selection in order to represent the structure of impairment, A&P limitation, contextual factors, and perceived health.

**Results:**

We find that impairment and A&P limitations are not directly related but only via a cluster of contextual factors. Similarly, impairment and perceived health are not directly related. On the other hand, perceived health is directly connected with A&P limitations. We hypothesize that contextual factors have a moderating and/or mediating effect on the relationship of impairment, A&P limitations, and perceived health.

**Conclusion:**

The disability paradox seems to dissolve when contextual factors are put into consideration. Contextual factors may be responsible for some persons with impairments developing A&P limitations and others not. In turn, persons with impairments may only then perceive bad health when they experience A&P limitation. Political interventions at the level of the environment may reduce the number of persons who perceive bad health.

## Background

According to the World Health Organization’s (WHO) recent World Report on Disability, over a billion people live with disabilities and accordingly represent over 15% of the world’s population [1]. This number is steadily increasing as the world’s health is compromised by increasing numbers of non-fatal injuries due to road traffic accidents [2,3] and disasters [4] as well as non-communicable disease [5]. These may lead to chronic health conditions and disabilities with which people may live for many years.

Following the WHO’s current bio-psycho-social model of disability described in the International Classification of Functioning, Disability and Health (ICF; cf. [6]), disability is not an attribute of individuals, but rather a set of difficulties individuals may experience in interaction with their social and physical environments [6,7]. Disability can be broken down into difficulties with functioning in different ICF components: impairments in *body functions and structures*, limitations in *activities*, and restrictions in *participation*. All of these components and the relations between them are further assumed to be affected by *contextual*, i.e. *environmental* and *personal*, *factors*[6]. The United Nations’ Convention on the Rights of Persons with Disabilities (article 29, b; cf. [8]) thus calls upon state parties to “promote actively an environment [social and attitudinal] in which persons with disabilities can effectively and fully participate in the conduct of public affairs”. Against this backdrop, it has also become a political imperative to research environmental factors’ impact on disability in order to come up with potential intervention targets [6].

In order to be able to address disability with appropriate interventions, we need to take this paradigm shift (i.e. introducing the contextual situation in a pivotal position) seriously and try to better understand the relationships between the components of disability. In this respect, studies have produced the seemingly surprising result, the so-called “disability paradox” introduced by Albrecht and Devlieger [9], that persons with severe impairments may nevertheless report high levels of quality of life (QoL) [1,10]. “In practice, the anomaly is that patients’ perceptions of personal health, well-being and life satisfaction are often discordant with their objective health status and disability” (see [9], p. 978). For example, the 2007-2008 Australian National Health Survey reports that 40 percent of people with a severe or profound impairment rate their health as good, very good, or excellent [11].

While Albrecht and Devlieger [9] explain this paradox with a balance theory framework (see [12]) considering an equilibrium of “body, mind, and spirit” (see [9], p. 978), we focus on contextual factors. We claim that the disability paradox may be resolved if one considers that “people with the same impairment can experience very different degrees and types of restrictions [in activity and participation], depending on the context”, i.e. environmental and personal factors (cf. [1], p. 22). To evaluate this theoretical conjecture, we need to assess data on all of the ICF models’ components independently and then explore interactions between them [6]. In order to do so, data from health surveys can be linked to the ICF components [13,14]. As the ICF does not provide separate lists for activities and participation we treat limitations in activities and participation (A&P limitations) as one component. In the ICF framework different components of functioning and contextual factors furthermore influence each other mutually. Consequently, many outcomes need to be considered at the same time.

In order to be able to study these multiple outcomes at one time, we reformulate the research problem in terms of conditional independence and then analyze it through a conditional independence graph [15]. We say that two events (or two variables) are independent if knowledge about one of the two does not influence the probability to observe the other one. Conversely, conditional independence implies that the probabilities of two events (or two variables) are independent if knowledge about additional events (or variables) is available. For instance, many wheelchair users may experience A&P limitations because they live in inaccessible neighborhoods. The connection between wheelchair and A&P limitation may disappear if we condition on neighborhood accessibility.

The objective of our study is to evaluate the role of contextual factors in the relationship of impairment, limitations in A&P, and perceived health (as one component of QoL). Therefore, our specific aims are to examine, against the background of contextual factors, the associations 1) between impairment, pain, and A&P limitation; 2) between impairment, pain, and perceived health; and 3) between A&P limitation and perceived health. In order to be able to address these aims simultaneously we apply conditional independence graphs.

## Methods

### Study Population

This study is a secondary analysis of cross-sectional data on functioning and disability from the Swiss Health Survey (SHS) in 2007 [16] (Bundesamt für Statistik, Schweizer Gesundheitsbefragung 2007). Data were obtained from the Federal Statistics Office of Switzerland. The original study was based on a stratified random sample of the Swiss general population aged 15 or older. A telephone survey was completed by a total of 18,760 persons, corresponding to a participation rate of 66 percent. The study was conducted by M.I.S.-Trend SA, Lausanne and Gümligen on behalf of the Swiss Federal Statistics Office. The data protection is ensured under the federal statistics law and data protection law. All data was treated highly confidential and anonymised [17].

### Measures

The SHS included various information on functioning, especially on pain, impairment, and A&P limitation. Since the respective items had different scale levels, we dichotomized them so that 1 was indicative of having some kind of problem. We then constructed sum indices for pain, impairment, and A&P limitation. Perceived health was measured with the following question and answer options: How would you rate your health in general? Very good, good, fair, poor, very poor. As personal factors we included the following demographics, health behaviors, and socio-economic status (SES). Demographics comprised gender, age, marital status. Health behaviors included alcohol consumption, current smoking, and regular leisure time physical activity (understood as lifestyle). Years of formal education, equivalent household income, paid employment, and migration background were used as indicators of individual level socio-economic status. For environmental factors, we created two sum indices for social network utilization and perceived social support, respectively. The built sum indices are illustrated in Table 1. On the macro- or cantonal (county) level, we obtained information on the cantonal Gross Domestic Products (GDP), Gini-Coefficients (which are a measure of income distribution ranging from complete equality expressed as 0, i.e. every person receiving the same amount of money, to complete inequality expressed as 1, i.e. a single person receiving all money), and crime rates for 2006.

**Table 1:** Sum scores

**Construct**	**Variable specification**
Impairment	Problems with …
	…vision
	…hearing
	…speaking
	…body mass index (i.e. over 30 or under 16)
	…urinary incontinence
	…defecation
	…feeling weak, tired, or a lack of energy
	…sleeping
	…tachycardia
	Range of sum index: 0-9
Pain	Pain in…
	…head
	…chest
	…stomach
	…back
	…hands
	…joints
	Range of sum index: 0-6
Activity &	Problems with independently…
participation	…walking
limitation	…eating
	…getting up from bed or chair
	…dressing
	…using the toilet
	…taking a shower or bath
	…preparing meals
	…using a telephone
	…doing the laundry
	…caring for finances/accounting
	…using public transport
	…doing major household tasks
	…doing shopping
	Range of sum index: 0-13
Social support	Having…
	…no feelings of loneliness
	…no feeling of missing someone to turn to
	…at least one supportive family member
	…someone to turn to
	Range of sum index: 0-4
Social network utilization	At least weekly…
	…visits from family
	…phone calls with family
	…visits from friends
	…phone calls with friends
	…participation in clubs/associations/parties
	Range of sum index: 0-5

Construction rules of sum indices for functioning (pain, impairment, activity and participation limitation) and social integration (social support and social network utilization) from 37 dichotomous (yes=1/no=0) variables. Reproduced from [15].

Across all variables used in the index construction or modeling, less than 0.85 percent of replies were missing. Imputation of missing values (i.e. estimating them from all observed data) did not affect the results reported here. We thus report on the unimputed data.

### Statistical Analysis

**Figure 1:**
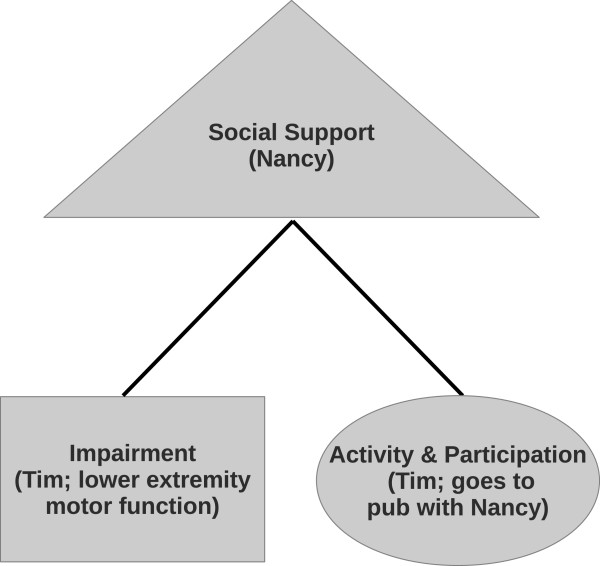
**Conditional independence graph.** The figure shows an example of a conditional independence graph. Due to the support of Nancy, Tim - who opperates in a wheelchar - is not limited in his participatation in common activities (e.g. going to a pub).

Most statistical analyses can be represented in terms of a graph. Nodes represent variables and edges reflect associations among variables. Conditional independence graphs (CIG), in particular, describe the association among any two nodes in the graph conditioned on the remainder of *p*−2variables. For example, Tim operates a wheelchair. However, he also has a lot of support from his wife Nancy. Thus, he does not experience limitations in A&P. This statement can be represented in terms of a CIG as shown in Figure 1. The three variables A&P limitation, supports, and impairment appear as nodes. The two nodes A&P limitation and impairment are only connected via a path of edges through supports. This structure reflects conditional independence of A&P limitation and impairment conditional on having appropriate supports.

We reproduce an analysis by Fellinghauer et al. [15] which focused on the question if all *p* = 20 variables are part of a single connected component. Here, however, we are mainly focusing on the constructs contextual factors, limitations in A&P, impairment, and perceived health and how they are connected. The graphical model we use is called Graphical Random Forests (GRaFo; cf. [15]) which can be used to model both discrete variables (e.g. gender) and continuous variables (e.g. age). The method is based on classification (for discrete outcomes) and regression (for continuous outcomes) trees (CART; cf. [18]). We regress each of the *p* variables on all remaining *p* − 1 variables (i.e. each variable is treated as outcome at some point). In order to avoid model overfitting (i.e. to avoid estimating parameters such that they are difficult to generalize to any other than the observed sample) we use random feature selection [19], i.e. predictors included in each model are randomly selected. This makes it necessary to draw sub-samples of the data (bootstrap; cf. [20]) in order to give every candidate predictor the same chance to be included in each of the models and to enhance reliability of the parameters. As a result we get a number of trees, i.e. a Random Forest [21]. Eventually, the GRaFo framework uses an algorithm to determine the most stable associations (stability selection; cf. [22]) and selects the most relevant edges/associations of variables to be included in the final graph. The general idea of GRaFo is to specify an upper bound on the expected number of false positives (i.e. false edges) R(V) that we are willing to accept in the worst case. According to this bound, edges that are sufficiently stable will be selected. Furthermore, GRaFo is conservative (with respect to achieving this bound) as a recent simulation study found that the observed false positive error is largely below the specified bound on false positives for *p* ≤ 100 [15].

In this study, we applied GRaFo for *p* = 20 and an upper error bound of R(V)≤5 false positives. We report for each edge from which bound on R(V) it is included in the graph (up to the maximum bound of size of 5). In general, smaller bounds on R(V) in the figure indicate more stable associations.

## Results

**Figure 2:**
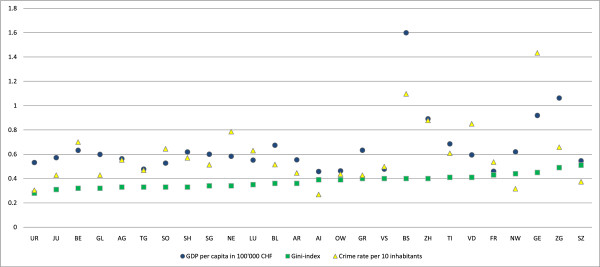
**Macro-level variables.** The figure shows the macro-level indicators across cantons.

A description of the study population is displayed in Table 2. We find that over 70% of the participants have at least one impairment and over 15% of the participants report one or more limitations in activity and participation. Specific impairments range from 42% reporting lack of energy and drive to 1.5% reporting problems with speaking; specific A&P limitations range from 12% reporting difficulties in accomplishing major household tasks to 0.5% reporting eating problems. Pain is most prevalent in the back (44%) and least prevalent in the chest (9%). Median perceived health equals “good”. Figure 2 shows the distribution of the macro-variables across the Swiss cantons which is rather similar with the exception of Basel city (BS), Geneva (GE), and Zug (ZG) which have comparably high per capita GDPs and/or crime rates. The Gini-coefficient ranges from around 0.25 in the county Uri to more than 0.5 in the neighbor county Schwyz.

**Table 2:** Individual-level variables

ICF Component	Total	
Functioning		
	Perceived health	
	*median* (90%Q; 10%Q)	2.0 (3.0; 1.0)
	Pain *mean* (SD)	1.6 (1.4)
	At least one pain problem, *n* (%)	14,166 (75.8)
	Impairment, *mean* (SD)	1.4 (1.3)
	At least one impairment, *n* (%)	12,458 (70.6)
	A&P limitation, *mean* (SD)	0.4 (1.4)
	At least on A&P limitation, *n* (%)	2,941 (15.8)
Personal Factors		
	Age *mean* (SD)	49.6 (18.5)
	Male *n* (%)	8,424 (44.9)
Lifestyles		
	Alcohol in grams per day,	
	*mean* (SD)	9.2 (15.4)
	Current smokers, *n* (%)	5,091 (27.2)
	Low physical activity, *n* (%)	4,775 (25.5)
Socio-economic		
status		
	Paid employment, *n* (%)	11,497 (61.3)
	Years of formal education,	
	*mean* (SD)	13.0 (3.5)
	Income, *mean* (SD)	4,154.1 (3,058.6)
	Foreign origin of at	
	least one parent, *n* (%)	5,128 (28.7)
Environment		
	Social support, *mean* (SD)	3.3 (0.8)
	Social networks, *mean* (SD)	3.2 (1.2)
	Married, *n* (%)	9,539 (50.9)

The table gives a description of all variables on the individual level in terms of mean and standard deviation (SD) or number of cases (n) and percent of sample (%). For perceived health we report the median and the 10% and 90% quantiles (Q).

**Figure 3:**
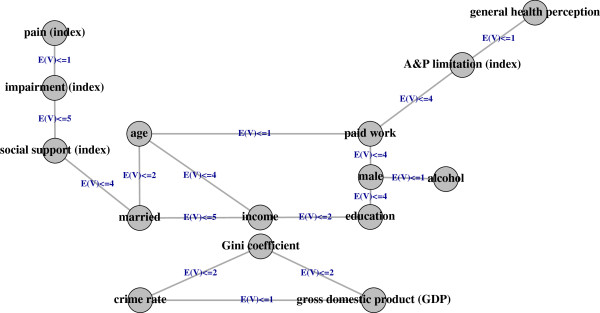
**Conditional independence graph.** The figure shows a conditional independence graph of the *p* = 20 variables (nodes) remaining after construction of indices based on the 2007 Swiss Health Survey estimated with GRaFo. Edges were selected with respect to an upper bound of 5 on the expected number of false positives R(V). For example, R(V)≤2 is to be interpreted as an edge which is present in a graph in which we expect up to 2 false edges. We cannot set this bound to 0 as this would be equivalent to a graph in which can be certain that all shown edges are correct (consequently, the algorithm would suggest an empty graph). Five nodes (social network utilization, migration background, smoker, work restriction, and leisure time physical activity) were isolated (no edges) and thus neglected. Reproduced from [15].

Figure 3 shows the resulting graph from our application of GRaFo to the data on functional health from the SHS with casewise deletion of missing values for a bound on the expected number of false positives R(V)≤5.

Impairment (including pain) in the top-left corner and limitations in activity and participation in the top-right corner are not directly connected, but only via the block of contextual factors in the middle (e.g. social support or being married). Impairment and perceived health are also not directly connected. Instead, every path between them contains both contextual factors and limitations in activities and participation. Limitations in activities and participation and perceived health are directly connected. There are two pathways leading from impairment to A&P limitation. One leads from social support via being married, income, education, and gender to paid work (socio-economic pathway). The other one leads from social support and being married to age and paid work (chronological pathway).

In the following, we will focus on the moderating and mediating role of contextual factors. A moderator may affect the strength and/or direction between a predictor and an outcome variable. A mediator on the other hand is affected by the predictor and accounts for the behavior of the outcome variable (see also [23]).

In terms of conditional independence, the upper path may be interpreted, with all other conditions held constant, as follows: impairment is independent of being married given perceived support, support is independent of age given marital status, paid work is independent of marital status given age (and at least one of the variables on the second pathway), and A&P limitations are independent of age given paid work. It follows that A&P limitations are independent of impairment given supports, marital status, age, and paid work which show a chain connection. This connection type alludes to a mediating relationship within the conditioning component, potentially starting from social roles related to age: older age makes it more likely to be married which makes social support more probable in turn; age moreover influences the likelihood of being in paid employment. We can hypothesize that married persons in employable age may be more often in paid employment and have less activity limitations in spite of impairments due to better supports.

The lower path may be interpreted, with all other conditions held constant, as follows: impairment is independent of marital status given support, support is independent of income given marital status, being married is independent of education given income and age, while income is independent of gender given educational level, age and marital status. Finally, educational level is independent of having employment and thus A&P limitation given gender and age of the person in question. Starting from sex and age roles, we may hypothesize that gender and age moderate the likelihood of having paid work while education and age moderate the impact of gender, having employment, and income on marriage and good supports which make it, in turn, less likely that impairments become A&P limitations.

The variables migration background, social network utilization, smoker, work restriction, and leisure time physical activity were neglected in the graph as they are not connected to any other node. Also, the three canton-level environmental factors form a separate component and are not related to the functional health component or any of the five neglected nodes.

## Discussion

This is the first study to apply a CIG to data on functioning and disability structured according to the new WHO disability model [6]. In that we tried to explain the so-called disability paradox that a relevant proportion of people with impairments reports good health and quality of life through conditioning on contextual factors, i.e. socio-economic determinants. We found that perceived health and A&P limitations are independent of impairment conditional on some of the considered contextual factors. An array of environmental and personal factors seems responsible for the translation of impairment or pain into A&P limitations (or vice versa). Health perception was still dependent on A&P limitations when conditioned on all contextual factors and other functioning domains in the model. For example, Tim, who uses a wheelchair, may have no activity limitations, because he has a lot of support from Nancy and access to many assistive technologies. Bob, on the other hand, is fairly isolated, does not find work because of his age, and may thus be limited in activities and participation in many areas.

**Figure 4:**
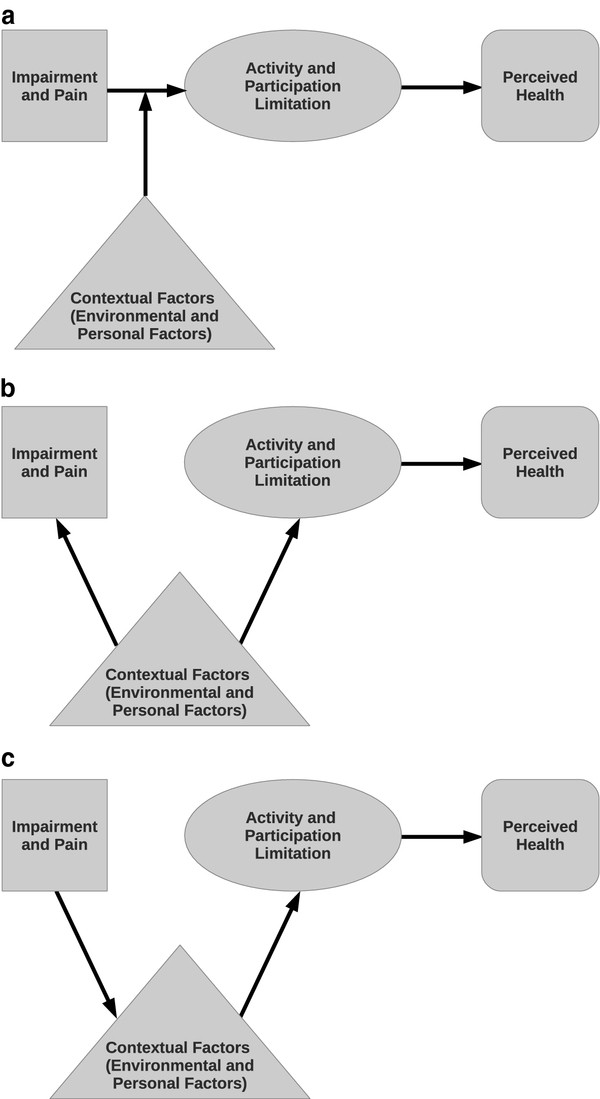
**Potential structures of the disability paradox depicted as directed graphs: Explanation (a) vs. Mediation (b) vs. Moderation (c) The figure shows potential structures of the disability paradox depicted as directed graphs.** More specifically, we formulate hypotheses on the relationship of limitations in activities and participation, contextual factors, impairment, and perceived health. Three different structures are suggested: explanation **(a)** vs. mediation **(b)** vs. moderation **(c)**. A moderator may affect the strength and/or direction between a predictor and an outcome variable. A mediator on the other hand is affected by the predictor and accounts for the behavior of the outcome variable [23]. As a special case of moderation we can regard explanation.

The findings thus support a central role of contextual factors in the moderation and mediation of the relationship between impairment and limitations in activity and participation. Figure 4 shows different possibilities to translate the found conditional independence relation into directed graphs: In the case of an explanation (Figure 4a), contextual factors determine both impairment and A&P limitations. The conditioning on contextual factors thus explains any correlation between impairment and A&P limitation. Mediation (Figure 4b), in turn, means that impairments influence contextual factors, e.g. decrease the socio-economic status of a person (drift), which then influences A&P limitations. In the moderation scenario (Figure 4c), the strength of the association between impairments and A&P limitations is influenced by contextual factors, e.g. in some contexts there might be no association of impairments and A&P limitations at all, while in others a strong relation might be found. Apart from social support, the other connecting variables may be viewed as personal factors in terms of ICF. However, they oftentimes imply an impact of external expectations as well [24], most evident in the case of gender and paid employment (also see the socio-economic pathway above). We thus tend to rather speak of contextual factors than differentiating environmental and personal factors as in the ICF, particularly against the background of the current data situation. For instance, environmental factors such as health services, assistive technology, rehabilitation program expenditure, and availability were not assessed in this study. We may thus only say, that some contextual factors pose an important contribution to solve the disability paradox at least with regard to the perceived health component of QoL. 

Surprisingly, we do not find any association of functioning, perceived health, personal factors, or micro-level (i.e. participant level) environmental factors with the macro-level indicators accounting for the clustering of the subjects in cantons. However, a time lag between macro-indicators and individual disability is possible (e.g. exposure to macro-level inequality at baseline may moderate disability at follow up) and has been shown elsewhere [25]. Also, transfers between cantons and social policy measures to redistribute income within the cantons and expenditure on rehabilitation services [26] were not considered in this study. Perhaps, social inequality on the macro-level may not play a major role in a country such as Switzerland, since it has an extremely high level of societal welfare as compared to many other countries. If we remove the three macro-level variables GDP, Gini, and crime rate from the model, the connectivity of the micro-level component does not change. In general, several candidate variables such as social network utilization did not appear in the graph. One reason may be our relatively conservative upper bound on the error that results in a graph which contains only very stable edges. On the other hand, this implies that we may miss some associations that were not as stable.

An important limitation of our study is the cross-sectional nature of the data that makes it impossible to draw conclusions on causality or even model feedback loops: For example, A&P limitation and impairment may reinforce each other through environmental and personal factors. There may also be an issue with our selection of variables that was restricted by the choices of the original survey team. Furthermore, our research is limited by missing values in the data. We cannot exclude the possibility that missing data occur not independently of levels of impairment, A&P limitation, or perceived health of the respondents. Assuming that observations are missing at random, an imputation did not change the resulting graph (not shown). In addition, results in perceived health may have been influenced by response shift [27]. However, evidence of response shift in disabled persons’ reporting of QoL is very weak [28].

A disadvantage of our method is the lack of a clear statement regarding the type of relationship among two nodes A and B. Neither do we know whether A causes B or vice versa, nor do we have information whether large values of A facilitate large values of B. The former information cannot be easily obtained from our framework as we only study associations (for causal graphs see e.g. [29]). The latter limitation arises mainly from computational limitations and future research may produce a version of GRaFo which can also provide this kind of information.

Also, GRaFo is based on certain technical assumptions [15] that are required to estimate conditional independence information but may be difficult to check in practice. However, given the high face validity of the findings and the achievement of control over false positives in a simulation study for a comparable mixed setting [15], the results of the GRaFo procedure seem satisfactory. Eventually, we only studied perceived health and not other components of QoL which leads to difficulties in comparing findings with the theory suggested by Albrecht and Devlieger [9]. However, we did not have data on life satisfaction and well-being.

Future research needs to develop more specific hypotheses about how environmental and personal factors interact in the disablement process. This presupposes that better measures of both personal [30] and environmental factors [31] as well as connecting pathways are established. Such pathways may assist the development of suitable interventions which avoid unintended side effects [32]. This is facilitated by the parallel study of the relation of multiple variables. In order to come up with suggestions on the policy level, the impact of macro and meso factors needs to be better understood and modeled. More fine-grained indicators than the ones used in our study need to be operationalized. On the country level, classifications of disability policies along a compensation and an integration component exist (see e.g. [33]). On the data collection level, the geographical linking of individual data to smaller units than cantons is desirable to better understand the macro-micro link. Conversely, better macro-level data, e.g. on the accessability of the surrounding neighborhood must be made publicly available by the statical offices in order to make meaningful research on the impact of policies on the lived experience of persons with disabilities [34] possible.

## Conclusion

Summarily, impairments do not necessarily lead to decreased perceived health if the translation of impairments into A&P limitations can be avoided. Similarly, impairments do not necessarily lead to A&P limitations. Modifiable environmental factors, such as social supports, moderate or mediate the relationship between body and activity and participation. Therefore, the number of persons with impairments who feel healthy and show high levels of performance in activity and participation may be increased with appropriate contextual interventions.

## Competing interests

The authors declare that there are no financial or non-financial competing interests.

## Authors’ contributions

Bernd Fellinghauer performed the statistical analyses and drafted the manuscript. Jan Reinhardt wrote the first draft, interpreted the results and drafted the manuscript. Gerold Stucki drafted parts of the manuscript. Jerome Bickenbach was involved in the interpretation of results, drafting of the manuscript, and provided general supervision. All authors have read and approved the final version of the manuscript.

## Pre-publication history

The pre-publication history for this paper can be accessed here:

http://www.biomedcentral.com/1471-2458/12/655/prepub

## References

[B1] WHO and The World Bank World Report on Disability 2011 Geneva: WHO Press

[B2] Ameratunga S Hijar M Norton R Road-traffic injuries: confronting disparities to address a global-health problem Lancet 2006 367 9521 1533 1540 16679167 10.1016/S0140-6736(06)68654-6

[B3] Peden M Hyder A Road traffic injuries are a global public health problem BMJ 2002 324 7346 1153 12003892 10.1136/bmj.324.7346.1153 PMC1123102

[B4] Reinhardt JD Li J Gosney J Rathore FA Haig AJ Marx M DeLisa JA International Society of Physical and Rehabilitation Medicine’s Sub-Committee on Rehabilitation Disaster Relief. Disability and health-related rehabilitation in international disaster relief Glob Health Action 2011 4 7191 10.3402/gha.v4i0.7191. Epub 2011 Aug 16 21866223 PMC3160807

[B5] Dans A Ng N Varghese C Tai ES Firestone R Bonita R The rise of chronic non-communicable diseases in southeast Asia: time for action Lancet 2011 377 9766 680 690 21269677 10.1016/S0140-6736(10)61506-1

[B6] WHO International Classification of Functioning, Disability and Health (ICF) 2001 Geneva: WHO Press

[B7] Bickenbach JE Chatterji S Badley EM Üstün B Models of disablement, universalism and the international classification of impairments, disabilities and handicaps Soc Sci Med 1999 48 9 1173 1187 10220018 10.1016/s0277-9536(98)00441-9

[B8] United Nations General Assembly Convention on the rights of persons with disabilities. Resolution 61/106 2006 Available from [www.un.org/esa/socdev/enable/conventioninfo.htm]

[B9] Albrecht GL Devlieger PJ The disability paradox: high quality of life against all odds Soc Sci Med 1999 48 977 988 10390038 10.1016/s0277-9536(98)00411-0

[B10] Watson N Well, I Know this is Going to Sound very Strange to you, but I don’t See myself as a Disabled Person: Identity and Disability Disability & Soc 2002 17 509 527

[B11] Australian Bureau of Statistics National Health Survey: Summary of Results, 2007-2008 2009 Canberra: Australian Bureau of Statistics

[B12] Lewin K Principles of Topological Psychology 1936 New York: McGraw-Hill Book Company

[B13] Fayed N Cieza A Bickenbach JE Linking health and health-related information to the ICF: a systematic review of the literature from 2001 to 2008 Disabil Rehabil 2011 33 21-22 1941 1951 21303198 10.3109/09638288.2011.553704

[B14] Cieza A Geyh S Chatterji S Kostanjsek N Üstün B Stucki G ICF linking rules: an update based on lessons learned J Rehabil Med 2005 37 4 212 208 16024476 10.1080/16501970510040263

[B15] Fellinghauer B Bühlmann P Ryffel M von Rhein M Reinhardt JD Stable Graphical Model Estimation with Random Forests for Discrete, Continuous, and Mixed Variables Preprint (available from arXiv.org hosted by Cornell University: 1109.0152v1) 2011

[B16] Storni M Enquêtes, sources: Enquête suisse sur la santé Office Fédéral de la Statistique, Neuchâtel. Espace de l’Europe 10, 2010 Neuchâtel. [http://www.bfs.admin.ch] 2011. [Available from http://www.bfs.admin.ch]

[B17] Graf E Rapport de méthodes. Enquête suisse sur la santé 2007. Plan d’échantillonnage, pondérations et analyses pondérées des données 2010 Neuchâtel: Office Fédéral de la Statistique,

[B18] Breiman L Friedman J Olshen R Stone C Classification and Regression Trees 1984 California: Wadsworth, Inc.

[B19] Amit Y Geman D Shape Quantization and Recognition with Randomized Trees Neural Comput 1997 9 1545 1588

[B20] Efron B Bootstrap Methods: Another Look at the Jackknife Ann Stat 1979 7 1 26

[B21] Breiman L Random Forests Mach Learn 2001 45 5 32

[B22] Meinshausen N Bühlmann P Stability selection (with discussion) J Roy Stat Soc B 2010 72 4 417 473

[B23] Baron RM Kenny DA The moderator-mediator variable distinction in social psychological research: Conceptual, strategic, and statistical considerations J Personality and Social Psychology 1986 51 1173 1182 10.1037//0022-3514.51.6.1173 3806354

[B24] Nagi S Sussman M Some conceptual issues in disability and rehabilitation Sociology and Rehabilitation 1965 Washington, DC: American Sociological Association 100 113

[B25] Gadalla TM Fuller-Thomson E Examining the lag time between state-level income inequality and individual disabilities: a multilevel analysis Am J Public Health 2008 98 12 2187 2190 18923110 10.2105/AJPH.2008.134940 PMC2636529

[B26] Wahrendorf M Siegrist J Schröder M, Hank K, Börsch-Supan A Working conditions in mid-life and participation in voluntary work after labour market exit The Individual and the Welfare State – Life Histories in Europe 2011 Berlin: Springer 179 188

[B27] Schwartz CE Andresen EM Nosek MA Krahn GL Response shift theory: important implications for measuring quality of life in people with disability Arch Phys Med Rehabil 2007 88 4 529 539 17398257 10.1016/j.apmr.2006.12.032

[B28] Amundson R Quality of Life, Disability, and Hedonic Psychology J Theor Soc Behav 2010 40 4 374 392

[B29] Colombo D Maathuis MH Kalisch M Richardson TS Learning high-dimensional directed acyclic graphs with latent and selection variables Ann Stat 2012 40 294 321

[B30] Geyh S Müller R Peter C Bickenbach JE Post M Stucki G Cieza A Capturing the psychologic-personal perspective in spinal cord injury Amer J Physical Med 2011 90 11 Suppl 2 S79 96 10.1097/PHM.0b013e318230fb68 21975679

[B31] Reinhardt JD Miller J Stucki G Sykes C Gray DB Measuring impact of environmental factors on human functioning and disability: a review of various scientific approaches Disabil Rehabil 2011 33 23-24 2151 65 21548824 10.3109/09638288.2011.573053

[B32] Stucki G Reinhardt JD Grimby G Organizing human functioning and rehabilitation research into distinct scientific fields. Part II: Conceptual descriptions and domains for research J Rehabil Med 2007 39 4 299 307 17468802 10.2340/16501977-0051

[B33] OECD Sickness, Disability and Work: Breaking the Barriers 2010 OECD Publishing

[B34] Stucki G Bickenbach JE Post M Developing epidemiologic studies of people’s lived experience: the Swiss Spinal Cord Injury Cohort Study as a case in point Amer J Physical Med 2011 90 11 Supppl 2 S1 4 10.1097/PHM.0b013e318230fe6a 21975672

